# Supplementary figures and images for: Humans account for cognitive costs when finding shortcuts: An information-theoretic analysis of navigation
Source: PLoS Comput Biol. 2023 Jan 6;19(1):e1010829. doi: 10.1371/journal.pcbi.1010829 (PMC9851521; doi:10.1371/journal.pcbi.1010829)

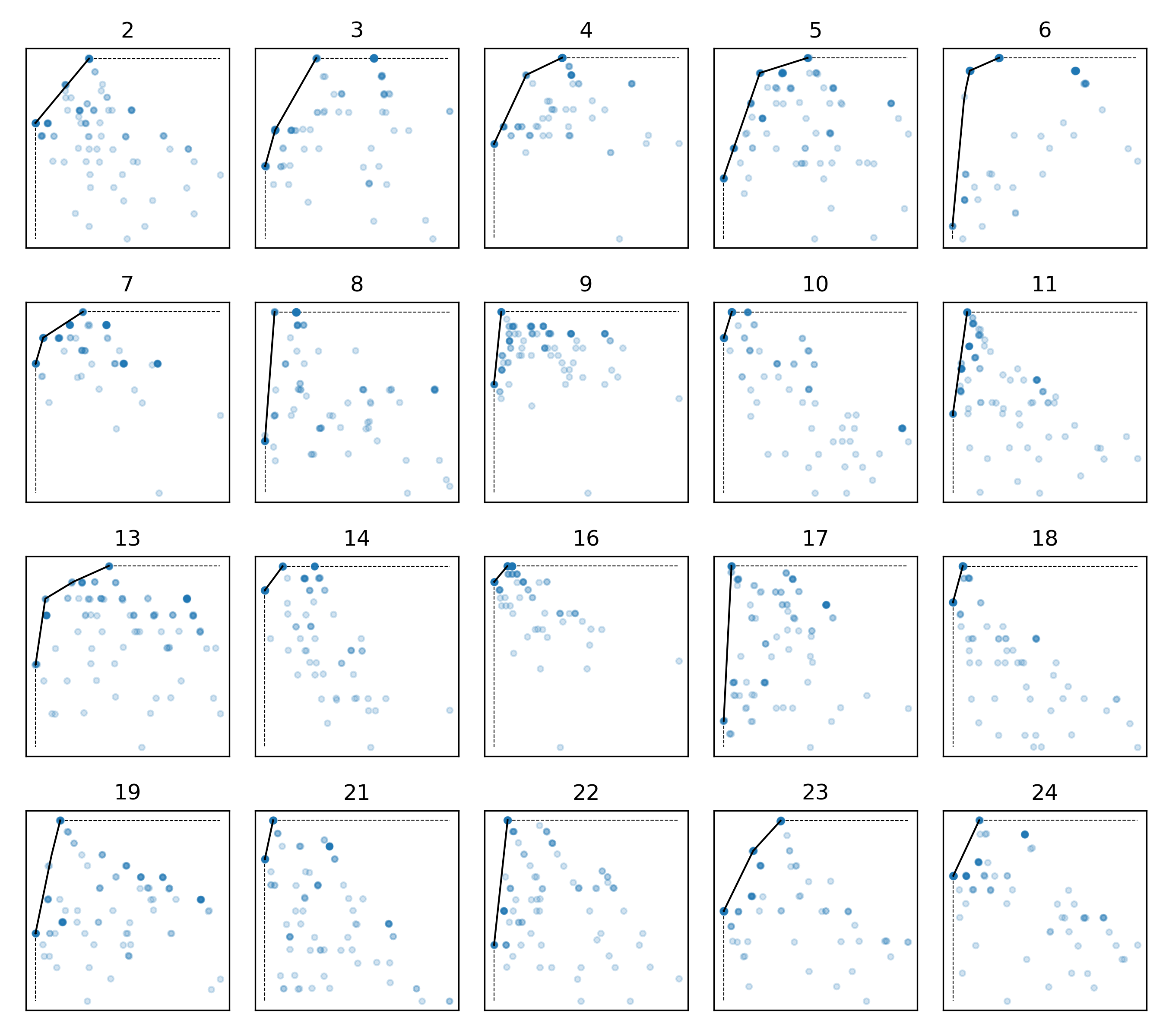

Supplement: S1 Fig — Reward / Control Information plots for the 20 pairs of start-goal locations collected by [33]. The blue points represent participants’ single trial data (darker blue means more points overlapping), solid black curve is the optimal curve, and together with the dashed black curve delimits the unachievable area (upper left portion of the plot). (TIFF) [file pcbi.1010829.s001.tiff]

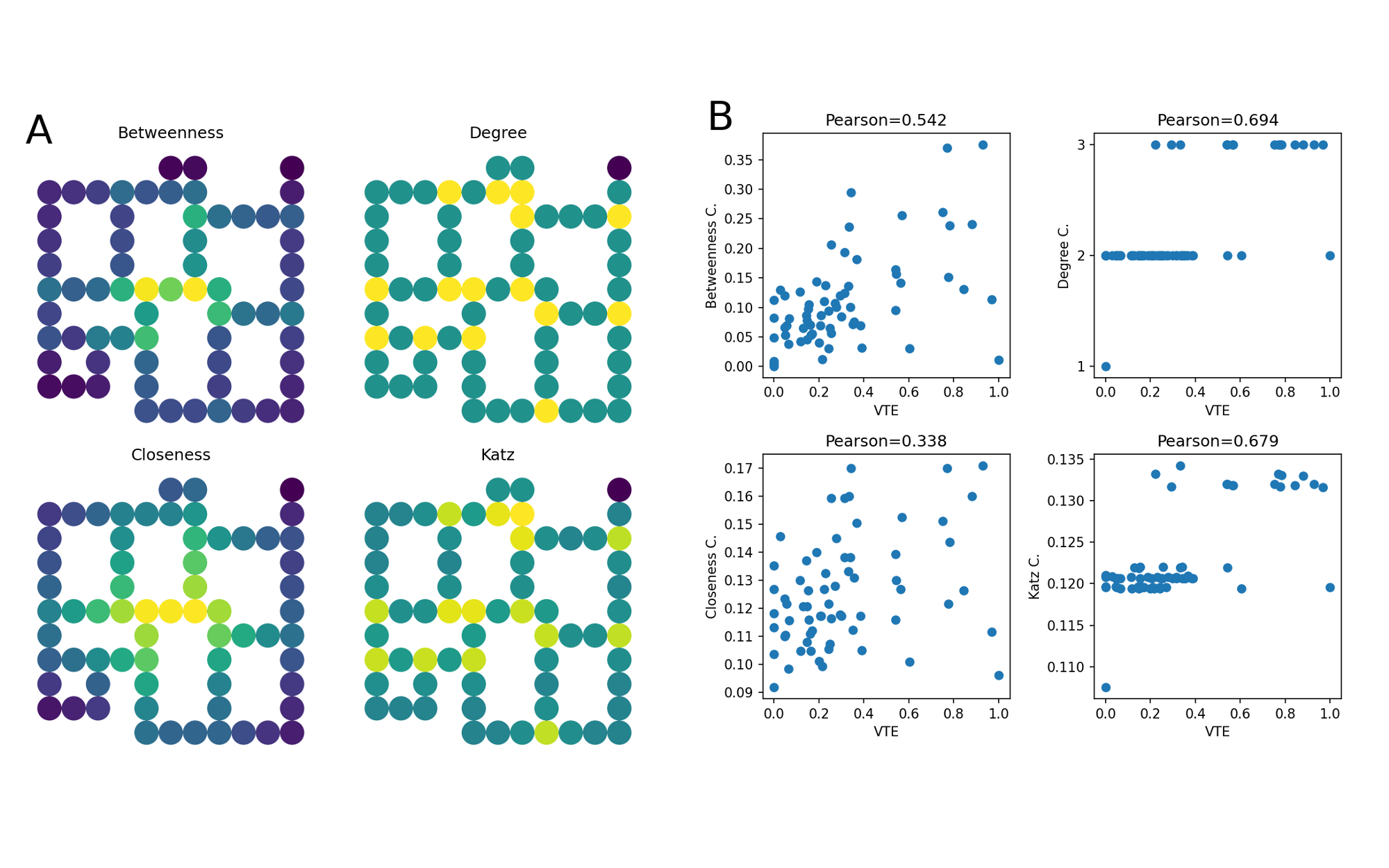

Supplement: S2 Fig — A) Betweenness, Degree, Closeness and Katz Centrality values for each state of the map; B) (Betweenness, Degree, Closeness and Katz) centrality value vs VTE value plots. (TIFF) [file pcbi.1010829.s002.tiff]

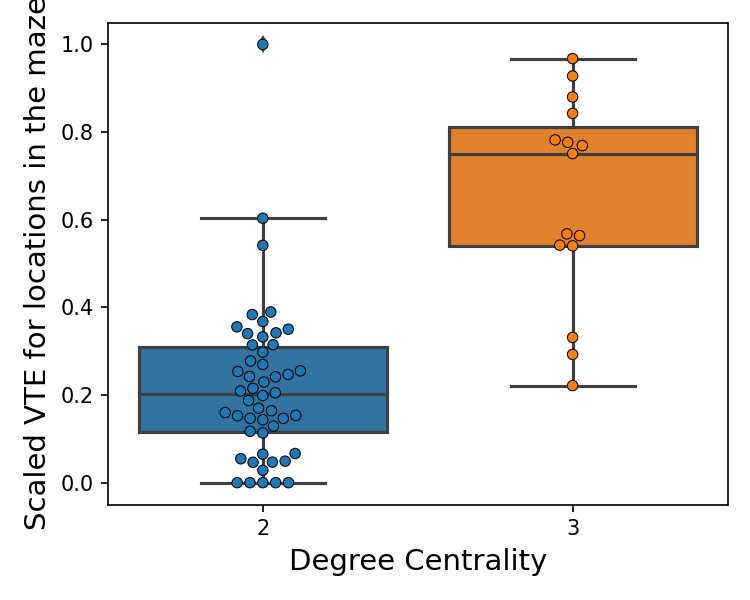

Supplement: S3 Fig — Scaled VTE for locations in the maze comparison for states with Degree Centrality equal to 2 and 3. A 2-sample Kolmogorov-Smirnov test confirmed (D = 0.74, p<0.001) that states with Degree Centrality equal to 3 show greater VTE values. (TIFF) [file pcbi.1010829.s003.tiff]

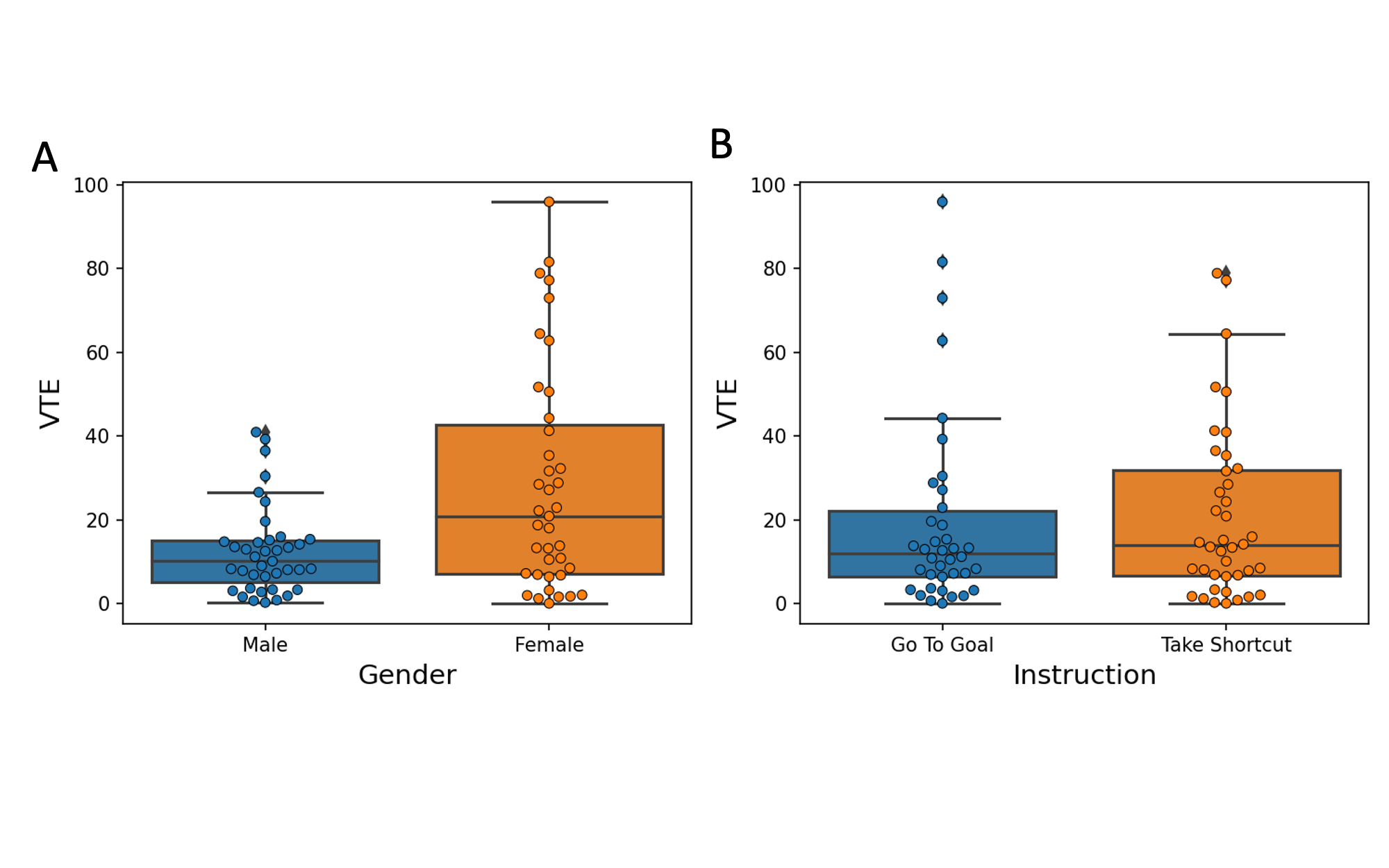

Supplement: S4 Fig — Left: Scaled VTE for locations in the maze compared by gender (D = 0.38, p = 0.006). Right: VTE values by condition (D = 0.16, p = 0.66). (TIFF) [file pcbi.1010829.s004.tiff]

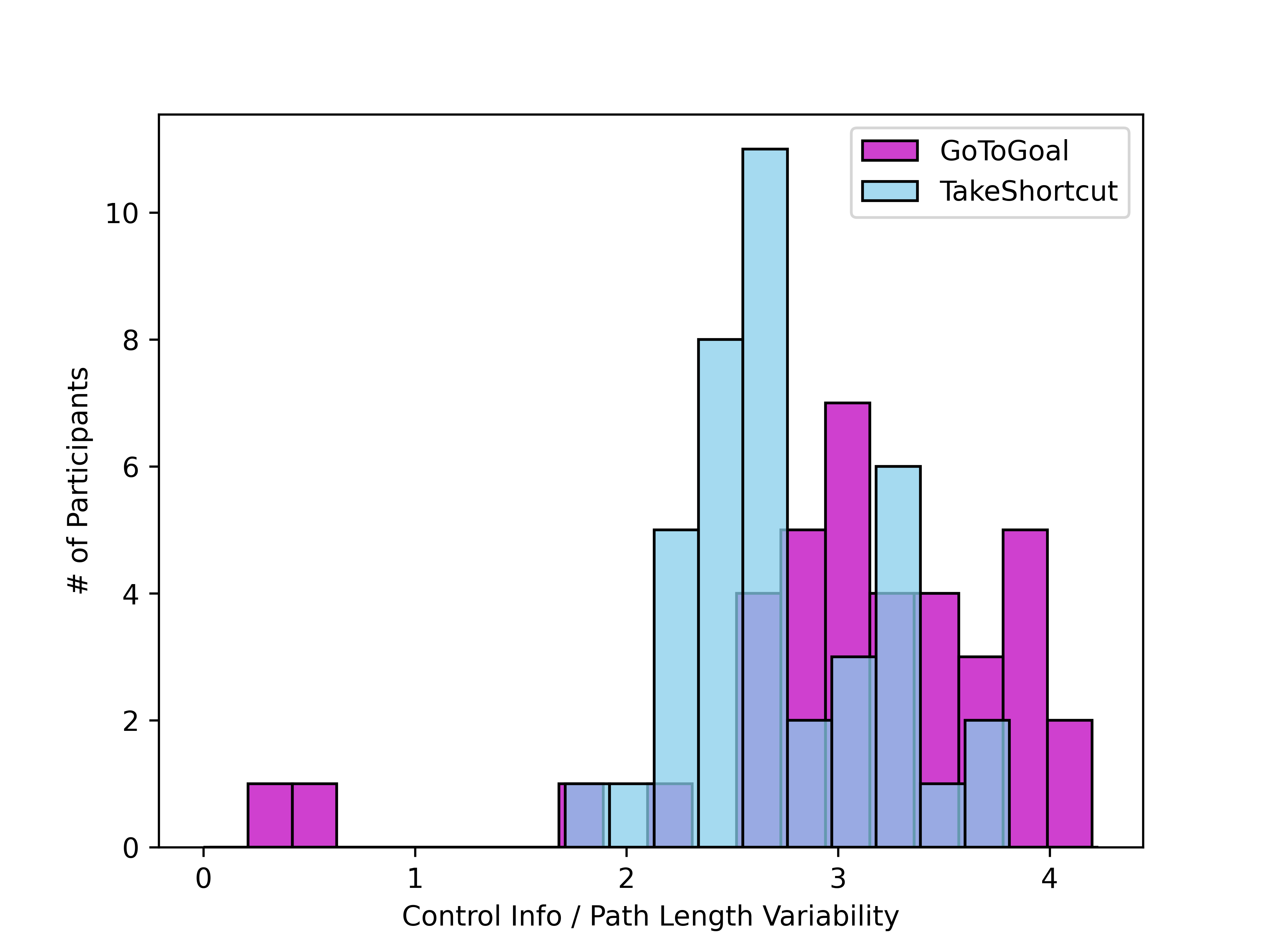

Supplement: S6 Fig — Histogram of the variability (i.e., standard deviation) of the Control Information–Path Length ratio across participants, as shown in Fig 7 (magenta: “Go To Goal” instruction, light blue: “Take Shortcut” instruction). (TIFF) [file pcbi.1010829.s006.tiff]
